# Prediction accuracy of regulatory elements from sequence varies by functional sequencing technique

**DOI:** 10.3389/fcimb.2023.1182567

**Published:** 2023-08-02

**Authors:** Ronald J. Nowling, Kimani Njoya, John G. Peters, Michelle M. Riehle

**Affiliations:** ^1^ Electrical Engineering and Computer Science, Milwaukee School of Engineering, Milwaukee, WI, United States; ^2^ Department of Microbiology and Immunology, Medical College of Wisconsin, Milwaukee, WI, United States

**Keywords:** enhancers, functional sequencing, machine learning, sequence models, DNase-seq, STARR-seq, ChIP-seq

## Abstract

**Introduction:**

Various sequencing based approaches are used to identify and characterize the activities of *cis*-regulatory elements in a genome-wide fashion. Some of these techniques rely on indirect markers such as histone modifications (ChIP-seq with histone antibodies) or chromatin accessibility (ATAC-seq, DNase-seq, FAIRE-seq), while other techniques use direct measures such as episomal assays measuring the enhancer properties of DNA sequences (STARR-seq) and direct measurement of the binding of transcription factors (ChIP-seq with transcription factor-specific antibodies). The activities of *cis*-regulatory elements such as enhancers, promoters, and repressors are determined by their sequence and secondary processes such as chromatin accessibility, DNA methylation, and bound histone markers.

**Methods:**

Here, machine learning models are employed to evaluate the accuracy with which *cis*-regulatory elements identified by various commonly used sequencing techniques can be predicted by their underlying sequence alone to distinguish between *cis*-regulatory activity that is reflective of sequence content versus secondary processes.

**Results and discussion:**

Models trained and evaluated on *D. melanogaster* sequences identified through DNase-seq and STARR-seq are significantly more accurate than models trained on sequences identified by H3K4me1, H3K4me3, and H3K27ac ChIP-seq, FAIRE-seq, and ATAC-seq. These results suggest that the activity detected by DNase-seq and STARR-seq can be largely explained by underlying DNA sequence, independent of secondary processes. Experimentally, a subset of DNase-seq and H3K4me1 ChIP-seq sequences were tested for enhancer activity using luciferase assays and compared with previous tests performed on STARR-seq sequences. The experimental data indicated that STARR-seq sequences are substantially enriched for enhancer-specific activity, while the DNase-seq and H3K4me1 ChIP-seq sequences are not. Taken together, these results indicate that the DNase-seq approach identifies a broad class of regulatory elements of which enhancers are a subset and the associated data are appropriate for training models for detecting regulatory activity from sequence alone, STARR-seq data are best for training enhancer-specific sequence models, and H3K4me1 ChIP-seq data are not well suited for training and evaluating sequence-based models for *cis*-regulatory element prediction.

## Introduction

*Cis*-regulatory elements (CREs) facilitate a variety of activities that modulate gene expression. Promoters and enhancers activate and increase gene expression, silencers decrease gene expression, and insulators separate topologically associating domains (TADs) and define regulatory boundaries. CREs contain transcription-factoring binding sites (TFBS) and other sequence patterns specific to their function that distinguish them from other parts of the non-coding genome, but the mechanistic details of their function including interactions between transcription factors are not well understood (Panigrahi and O’Malley, 2021). An increased understanding of CRE identity and function will increase our mechanistic understanding of how gene expression is controlled and potentially allow experimental or pharmacologic control of gene expression in the context of human disease or vector borne disease. Evidence from genome wide association studies has shown that genetic variation segregating within in CREs is linked to phenotypic variation, including in the context of human diseases such as prostate cancer, breast cancer, systematic lupus, Crohn’s disease, and inflammatory bowel disease (IBD) (Corradin and Scacheri, 2014; Williams et al., 2019; Nasser et al., 2021). Greater understanding of the function of CREs and impact of genetic variation coupled with greater efficiency of CRE identification could have significant clinical impacts.

Given the lack of a highly regular amino acid-like code for CREs, efforts to identify CREs are challenging. CREs are directly identified or inferred using several next generation sequencing (NGS) technologies that employ different indirect and direct approaches (Shlyueva et al., 2014; Tsompana and Buck, 2014; Sun et al., 2019). ATAC-seq, DNase-seq, and FAIRE-seq identify regions of open chromatin, through use of a transposase that inserts into open chromatin, an enzyme that digests DNA at open chromatin, or using formaldehyde fixation to separate nucleosome associated DNA from non-nucleosome depleted DNA, respectively (Song and Crawford, 2010; McKay and Lieb, 2013; Buenrostro et al., 2015). These regions of open chromatin represent chromosomal locations with enriched numbers of active regulatory elements. ChIP-seq uses antibodies to identify modified histones such as H3K4me1 (a histone modification associated with poised enhancers), H3K4me3 (a histone modification associated with promoters), and H3K27ac (a histone modification associated with active enhancers) that are associated with different regulatory activities (Heintzman et al., 2009; Creyghton et al., 2010; Ernst et al., 2011; Rada-Iglesias et al., 2018). Unlike the other methods, STARR-seq and its variant UMI-STARR-seq are ectopic, plasmid based assays that directly measure enhancer activity (Arnold et al., 2013; Neumayr et al., 2019). These assays are removed from chromatin context and facilitate the detection of any sequence with enhancer potential though cellular enhancer activity will be dynamic and vary by cell or tissue type, development time etc.

The genome-wide CRE maps generated by these -omics based approaches enable a number of downstream analyses and validation. Individual CREs can be PCR amplified, cloned and screened for the ability to modulate gene expression levels using a gold standard luciferase assay which queries enhancer activity in an ectopic assay, in the absence of chromatin (Arnold et al., 2013). Transcription factor binding sites (TFBSs) can be identified computationally through searches for motif patterns, either those conserved in related organisms and available through public databases such as JASPAR (Castro-Mondragon et al., 2022) or enriched in CREs compared with the remainder of the genome (Bailey, 2021). Genetic variants that alter motif sequences, particularly at conserved sites within TFBS motifs, can be identified and their individual impact on activity characterized (Jin et al., 2016; Yang et al., 2022). Lastly, examining CREs through an evolutionary lens can allow losses and gains of CREs associated with phenotypic differences between species to be identified (Stark et al., 2007; He et al., 2011; Arnold et al., 2014).

While next generation sequencing approaches are now widely employed, knowledge around the capabilities and limitations of using sequencing based approaches to identify CREs are still evolving. For example, H3K4me1 is associated with enhancers, and H3K4me3 is associated with promoters (Heintzman et al., 2009; Ernst et al., 2011). New evidence suggests, however, that the interpretation of these methylation and acetylation patterns may not be as straight-forward as initially thought. A recent study observed enrichment of H3K4me3 and depletion of H3K4me1 in highly active enhancers (Henriques et al., 2018). While the categorization of CREs into discrete classes provides analytical framework, this evidence suggests regulation of gene expression by CRE is complex.

Machine learning techniques provide a complementary approach to augment available experimental data addressing the identification and functional characterization of CREs. Machine learning efforts include *in silico* identification of CREs based solely on nucleotide sequence (Kazemian et al., 2011; Lee et al., 2011; Ghandi et al., 2014; Lee et al., 2016; Chen et al., 2018; Asma and Halfon, 2019; Koo and Eddy, 2019; Ni and Su, 2021; Butt et al., 2022; Ni et al., 2022), using latent factors to predict CRE activity across distinct cell types from sparse sampling (Schreiber et al, 2020a), predicting the impact of non-coding variants on regulatory elements (Zhou and Troyanskaya, 2015; Kelley et al., 2016; He et al., 2018), and predicting the impact on gene expression (Hafez et al., 2017; Kelley, 2020; Avsec et al., 2021). Despite these widespread efforts to computationally inform experimental work on CREs, the strengths and weaknesses of data derived from alternate next generation sequencing approaches and their impact on machine learning models have not been systematically examined.

The differences in the type of data generated by the array of sequencing methods used to identify and characterize CREs and how these differences propagate into the resulting models have yet to be sufficiently considered by the computational modeling community. Here this knowledge gap is addressed by training and evaluating machine learning models on chromatin accessibility (ATAC-seq, DNase-seq, FAIRE-seq), histone modification ChIP-seq (H3K4me1, H3K4me3, and H3K27ac), and direct measures of enhancer activity (STARR-seq and UMI-STARR-seq) assay data from *D. melanogaster*. Across this diverse set of experimental methods, substantial differences in accuracy were observed, indicating that the amount of signal variation explainable by sequence pattern alone varies across the sequencing methods. Randomly-sampled H3K4me1 and DNase peak sequences were experimentally tested for enhancer activity using luciferase assays and compared with similar published data from STARR-seq. Combined with computational analyses, we conclude that STARR-seq, UMI-STARR-seq, and DNase-seq demonstrate substantial benefits for CRE modeling based solely on nucleotide sequence. Observed differences in the specificity of (UMI) STARR-seq and DNase-seq for enhancers and broader regulatory elements, respectively, impact downstream models; the appropriateness of each type of data for each machine learning needs to be clearly communicated with end users.

## Materials and methods

### Data sets, preparation, and peak calling

Analyses were performed on chromatin accessibility (ATAC-seq, DNase-seq, FAIRE-seq), histone modification ChIP-seq (H3K4me1, H3K4me3, and H3K27ac), and direct enhancer activity reporter via an ectopic plasmid based assay (STARR-seq) data sets generated from experiments in *D. melanogaster* (see **Table 1** for references and accession numbers). The ATAC-seq and FAIRE-seq data sets were generated using wandering third instar larvae eye antennal imaginal disc tissue extracted from the FRT82 stock (Davie et al., 2015), while all other data sets were generated from *Drosophila melanogaster* S2 cells (Arnold et al., 2013; Henriques et al., 2018; de Almeida et al., 2022).

**Table 1:** Data sets and associated data processing parameters.

Data Set	Source	Tissue Type	Read Length (bp); Single (SE) or Paired (PE) End	Trimmomatic Settings	Control Data	Additional MACS Settings
ATAC-seq (FRT82 stock)	Davie et al., 2015	eye antennal imaginal discs	51 bp; SE	ILLUMINACLIP : TruSeq3-SE.fa:2:30:10 LEADING:3 TRAILING:3 SLIDINGWINDOW:4:15 MINLEN:36		-f BAM –nomodel –extsize 50
DNase-seq	Arnold et al., 2013	S2 cells	36 bp; SE	LEADING:3 TRAILING:3 SLIDINGWINDOW:4:15 MINLEN:20		-f BAM
FAIRE-seq (FRT82 stock)	Davie et al., 2015	eye antennal imaginal discs	50 bp; SE	ILLUMINACLIP : TruSeq3-SE.fa:2:30:10 LEADING:3 TRAILING:3 SLIDINGWINDOW:4:15 MINLEN:36		-f BAM –nomodel –extsize 50
H3K27ac ChIP-seq	Henriques et al., 2018	S2 cells	51 bp; PE	ILLUMINACLIP : TruSeq3-PE.fa:2:30:10 LEADING:3 TRAILING:3 SLIDINGWINDOW:4:15 MINLEN:36		-f BAMPE
H3K4me1 ChIP-seq	Henriques et al., 2018	S2 cells	51 bp; PE	ILLUMINACLIP : TruSeq3-PE.fa:2:30:10 LEADING:3 TRAILING:3 SLIDINGWINDOW:4:15 MINLEN:36		-f BAMPE
H3K4me3 ChIP-seq	Henriques et al., 2018	S2 cells	51 bp; PE	ILLUMINACLIP : TruSeq3-PE.fa:2:30:10 LEADING:3 TRAILING:3 SLIDINGWINDOW:4:15 MINLEN:36		-f BAMPE
STARR-seq (DSCP)	Arnold et al., 2013	S2 cells	36 bp; PE	LEADING:3 TRAILING:3 SLIDINGWINDOW:4:15 MINLEN:20	Yes	-f BAMPE
UMI-STARR-seq (DSCP)	de Almeida et al., 2022	S2 cells	36 bp; PE	ILLUMINACLIP : TruSeq3-PE.fa:2:30:10 LEADING:3 TRAILING:3 SLIDINGWINDOW:4:15 MINLEN:20	Yes	-f BAMPE

Sequencing reads were downloaded from the NIH SRA, cleaned and trimmed using Trimmomatic v0.39 (Bolger et al., 2014), and aligned to the *D. melanogaster* r6.45 genome (Hoskins et al., 2015) using BWA (bwa aln, default settings) v0.7.17-r1188 (Li and Durbin, 2009). The *D. melanogaster* genome was downloaded from Fly Base (Gramates et al., 2022). Aligned reads were filtered for mapping quality (-q 10 -F 0x0200 -F 0x0100 -F 0x004) using SAMTools v1.11 (using htslib v1.11-4) (Li et al., 2009). Peaks were called using MACS2 v 2.2.7.1 (Zhang et al., 2008) with FDR correction (-q 0.01) and the preset *D. melanogaster* genome size (-g dm). The data sets varied in terms of read lengths, single-end or paired-end, and the availability of control data, so parameters were adjusted appropriately. MACS peak calling parameters for ATAC-seq and FAIRE-seq data were taken from Davie et al. (2015). The parameters used for each data set are given in **Table 1**. The called peaks for each of the 8 data sets are available from Zenodo (Nowling, et al. 2023).

### Peak characterization

Genomic distributions of the peaks identified for each data set were examined. Based on genome annotations, exon and intron boundaries were written to BED files. Transcription start sites (TSSs) were defined as regions extending 500 bp upstream of protein coding sequence (corrected for strand orientation). Intergenic regions were defined by subtracting the exon, intron, and TSS regions from the overall chromosomes using BEDTools subtract (Quinlan and Hall, 2010; Quinlan, 2014). Coordinates of regions intersecting the peaks and each type of genomic element were calculated using BEDTools intersect. Coverage of each genomic element was normalized by dividing the summed lengths of the intersection regions by the summed lengths of the genomic element regions.

### Sequencing depth profiles

Read-depth profiles for the ATAC-seq, ChIP-seq DNase-seq, and FAIRE-seq data were generated around the STARR-seq peak centers using deepTools2 v3.5.1 (Ramírez et al., 2016). The filtered BAM files were combined into a single BAM file for each data set. BigWig files were generated for each data set using the bamCoverage tool with the parameters “–binSize 20 –normalizeUsing BPM –smoothLength 60 –extendReads –centerReads –ignoreDuplicates -e 114”, except for FAIRE-seq in the which the parameters “–binSize 20 –normalizeUsing BPM –smoothLength 60 –extendReads 150 –centerReads –ignoreDuplicates” were used. Matrices were generated using computeMatrix referencePoint command with the parameters “–referencePoint center -b 1000 -a 1000 –skipZeros”. Lastly, plots were generated using the plotProfile command.

### Sequence models

For each next generation assay, a sequence data set was constructed. Fore CREs, 501-bp sequences centered at the peak summits were extracted. A corresponding 501-bp control sample was generated for each peak using BEDTools shuffle with exclusions for coding sequences and any of the peaks from the corresponding data set. Only peaks on the 2L, 2R, 3L, 3R, and X chromosomes were used. Peak and control sequences were assigned positive and negative labels, respectively.

Logistic regression models were trained to distinguish between peak and control sequences. For each sequence, all k-mers from 6 to 8 nucleotides were identified and counted using CountVectorizer(ngram_range=(6, 8), analyzer=“char”) from Scikit-learn (Pedregosa et al., 2011). Scikit-learn’s CountVectorizer identified 6-8-mers present in the training data. If a particular 6-, 7-, or 8-mer was not present in the training data, it was not included in the resulting vocabulary. K-mers present in the target (testing) but not training data were ignored. Reverse complements of k-mers were not explicitly calculated. Models were evaluated using a cross-fold validation scheme in which the sequences were partitioned into folds by chromosome (five folds total; Schreiber et al., 2020b). An ensemble of 48 L2-regularized logistic regression models were trained using SGDClassifier(loss=“log_loss”, penalty=“l2”, alpha=0.01, max_iter=1000, shuffle=True) and BaggingClassifier(bootstrap=False, bootstrap_features=False) from Scikit-learn. Training for each logistic regression model was initialized with a different random seed. The probability of being derived from a next generation assay peak was estimated for each sequence. Predictions were evaluated using Receiver Operator Characteristic (ROC) area under the curve (AUC) calculated using the auc_roc_score() function from Scikit-learn.

### Validation by luciferase reporter assays 

#### Amplification and cloning of test fragments

The peak sequences and controls were PCR-amplified from genomic DNA isolated from Schneider 2 (S2) cells; a cell line originally derived from late embryonic stage *Drosophila melanogaster* embryos (Schneider, 1972). The PCR primers used for each genomic region were designed with Kpn1 (5’ TAGAGGTACC) and Sac1 (5’ GCTAGAGCTC) restriction sites at the 5’ end to allow for restriction enzyme cloning plus four additional bases at the 5’ ends to increase digestion efficiency. All primer sequences are available as **Supplementary Table 1** in the **Supplementary Information**. PCR amplification was performed in reactions consisting of 1X Ultra Mix (PCR Biosystems), 250 nM forward and reverse primers each (IDT), and 20 ng S2 genomic DNA. Cycling conditions were initial denaturation at 98°C for 30 sec, followed by 30 cycles of 98°C for 10 sec, 60°C for 30 sec and 72°C for 45 sec followed by final extension at 72°C for 45 sec. The PCR amplicons were cleaned over columns (IBI Scientific). PCR amplicons and 2.5 µg firefly luciferase reporter vector, pGL3-Gateway-DSCP (AddGene 71506) (Arnold et al., 2013), were double digested with 20 U Kpn1-HF and 20 U Sac1-HF (New England Biolabs) in 1X rCutSmart Buffer (New England Biolabs) at 37°C for 1 hour. The linearized firefly luciferase reporter vector was dephosphorylated by incubation with 1 U calf intestinal alkaline phosphatase (CIAP)(Invitrogen) at 37°C for 5 min. The CIAP was then inactivated with 4 mM EDTA and incubated at 65°C for 15 min. The digested PCR amplicons and luciferase reporter vector were subjected to column cleanups (IBI Scientific). The digested luciferase vector and PCR amplicons were combined at a mass ratio of 1:5 and ligated using 5 U T4 DNA ligase (Thermo Scientific) in 1X T4 DNA ligase buffer (Thermo Scientific) at 22°C for 1 hour followed by heat inactivation at 70°C for 5 min. The ligation reaction was transformed into OneShot OmniMax 2T1 chemically competent *E. coli* cells (Invitrogen) and plated on LB agar with ampicillin. Individual colonies were picked into 10 µl DNase/RNase-free distilled water (Invitrogen) and screened for correct insert size by PCR reactions containing 1X Ultra Mix (PCR Biosystems), 250 nM RVprimer3 forward primer (5’ CTAGCAAAATAGGCTGTCCC), 250 nM LucNrev reverse primer (5’ CCTTATGCAGTTGCTCTCC) and 2 µl water culture. Cycling conditions were initial denaturation at 95°C for 10 min, followed by 30 cycles of 95°C for 15 sec, 55°C for 30 sec and 72°C for 45 sec and a final extension at 72°C for 7 min. For transformants with the correct insert size, overnight cultures were prepared in LB medium containing ampicillin and subjected to plasmid purification (IBI Scientific). The clones were Sanger sequenced to verify insert sequence and sequences are provided in the **Supplementary Materials**.

#### Measurement of enhancer activity by luciferase assay

S2 cells at 80% confluency were counted on a hemocytometer, seeded in 96 well plates at 25,000 cells/well in 65 µl Schneider’s medium and incubated for 24 h at 27°C. Transfections were performed using Lipofectamine 3000 (Invitrogen) and 2 reporter vectors: renilla luciferase control vector pRL-ubi-63E (AddGene 74280) and the firefly luciferase vector pGL3-Gateway-DSCP with cloned candidate peak sequences/controls inserts at a ratio of 1:80 (1.125 ng renilla vector and 90 ng firefly vector). To monitor assay consistency and performance, all test plates contained cells that were transfected with a positive control fragment that was previously tested and found to have enhancer activity (Gohl et al., 2008) and a negative control fragment without enhancer activity. Negative control fragments were size- and location-matched in regions of the genome that did not overlap DNase or ChIP-seq peaks. Following transfection, plates were agitated at 350 rpm for 30 s on a MixMate (Eppendorf) and incubated for 24 h at 27°C. To determine the enhancer activity of cloned fragments, the Dual-Glo Luciferase Assay System (Promega) was used following the supplier protocol. Luminescence was measured on a GloMax Discover instrument (Promega). Firefly luminescence was measured after addition of the Dual-Glo reagent and a 20-minute incubation, and renilla luminescence was measured subsequently after the addition of Stop & Glo reagent and a second 20-minute incubation. All samples were tested in 6-fold technical replication. To quantify activity, firefly luciferase luminescence measurements were normalized to the renilla luciferase measurements for the same technical replicate/well. Peak fragment activity was expressed relative to the normalized activity of the negative control. Activity for the test fragments was compared to an activity of 1 (the normalized activity level of the negative control fragment) using a one sample T-test. A significance threshold of 0.05 with a Bonferroni correction was used for hypothesis testing.

## Results and discussion

### Peak calling with a common genome version

**Figure 1:**
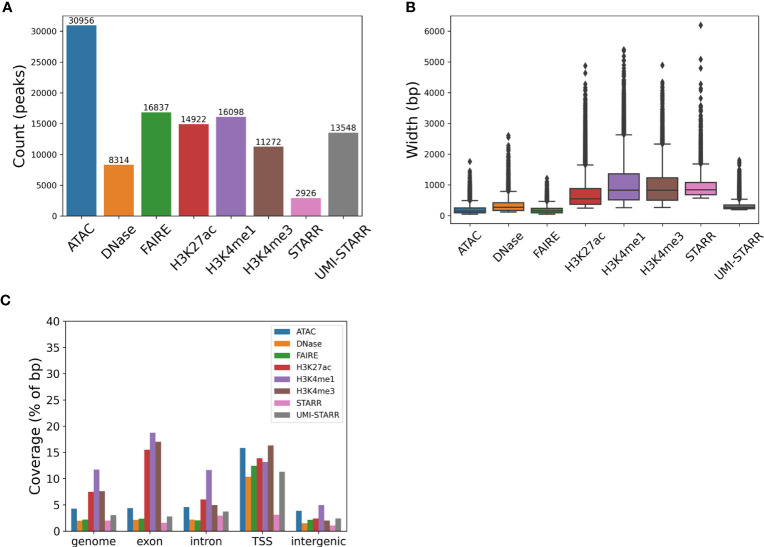
Characterization of peaks from sequencing data sets generated in *Drosophila melanogaster*. **(A)** The number of peaks called for each data set. **(B)** The widths (in bp) of peaks called for each data set. **(C)** Coverage (percentage of total bp) of the genome and its annotated exons, introns, transcription start sites (TSSs), and intergenic (sequence in between annotated genes).

Peaks were called for each of eight data sets (see **Table 2**); three chromatin accessibility data sets, H3K4me1, H3K4me3, and H3K27ac histone modification ChIP-seq data sets, and two direct assay activity data sets (**Figure 1**; **Table 2**). The number of called peaks ranged from 2,926 (STARR-seq) to 30,956 (ATAC-seq, **Figure 1A** and **Table 2**) with average peak widths from 185 bp (FAIRE-seq) to 1,044 bp (H3K4me1, **Figure 1B** and **Table 2**) and total genome coverage of 2.0% (multiple data sets) to 11.7% (H3K4me1, **Figure 1C** and **Table 2**).

**Table 2:** Peak characteristics.

Data Set	Source	Peak Count	Peak Width (bp)	Genome Coverage
ATAC-seq (eye antennal imaginal discs, FRT82 stock)	Davie et al., 2015	30,956	199 ± 156	4.3%
DNase-seq (S2 cells)	Arnold et al., 2013	8,314	343 ± 305	2.0%
FAIRE-seq (eye antennal imaginal discs, FRT82 stock)	Davie et al., 2015	16,387	185 ± 127	2.2%
H3K27ac ChIP-seq (S2 cells)	Henriques et al., 2018	11,272	722 ± 519	7.5%
H3K4me1 ChIP-seq (S2 cells)	Henriques et al., 2018	14,922	1,044 ± 717	11.7%
H3K4me3 ChIP-seq (S2 cells)	Henriques et al., 2018	16,098	968 ± 609	7.6%
STARR-seq (S2 cells, DSCP)	Arnold et al., 2013	2,926	988 ± 490	2.0%
UMI-STARR-seq (S2 cells, DSCP)	de Almeida et al., 2022	13,548	325 ± 157	3.1%

### Sequence-activity association varies across the sequencing methods

Machine learning models were evaluated on their ability to distinguish experimentally identified peak sequences from non-coding, non-peak, control sequences randomly sampled from across the genome. One set of models was created for each sequencing data set. Model prediction performance can be interpreted as a measure of the association between sequence patterns and the observed activity (e.g., as measured by a particular sequencing method). High prediction accuracies indicate that the sequence patterns completely or mostly explain differences in observed activity, while low prediction accuracies may be due to confounding factors (e.g., location of the peaks relative to the active part of the sequence or secondary processes such as suppressed activity due to methylation).Since the activity of regulatory elements is partly a function of their sequence (e.g., transcription factor binding sites), we hypothesized that the association between DNA sequence and CRE activity would be high across all of the data sets.

**Figure 2:**
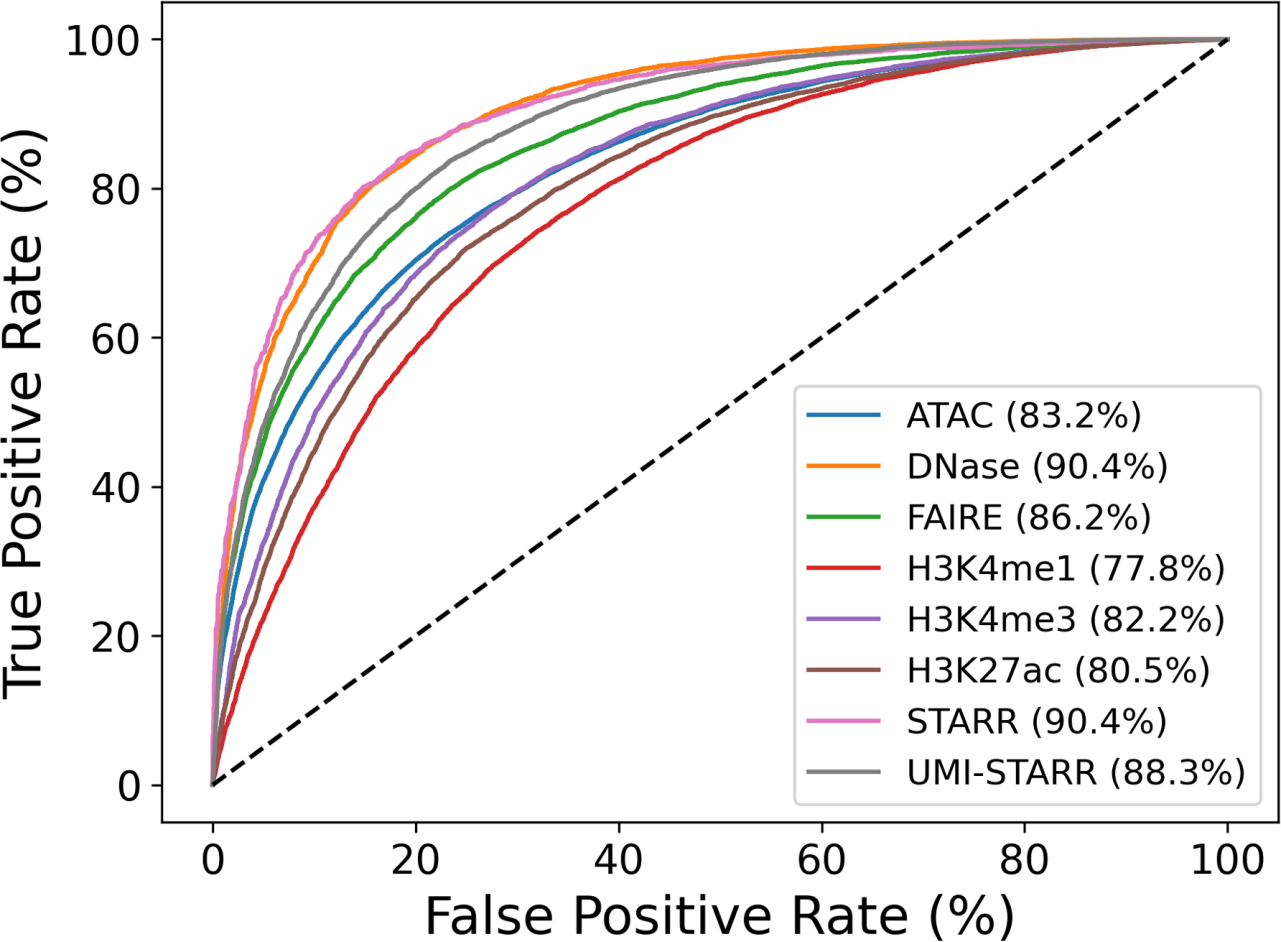
Evaluation of sequences models trained to distinguish peak from control sequences. Peak sequences from 2 each data set were divided by chromosome arm, 2L, 2R, 3L, 3R, and X. An equal number of non-overlapping control sequences were randomly sampled. Ensembles of 48 logistic regression models using counts of 6-mers to 9-mers were trained and evaluated and to distinguish peak sequences from control sequences. Training and evaluation were performed using five-fold cross-fold validation (one fold per chromosome arm). Prediction accuracies were evaluated by computing receiver operator characteristic (ROC) curves and the associated areas under the curves (AUC).

Contrary to our expectations, model prediction performance (measured by ROC AUC) differed substantially across the data sets, varying from 77.8% for H3K4me1 data to 90.4% for DNase-seq and STARR-seq data (**Figure 2**). Accuracy as measured by ROC AUC was significantly negative correlated (p<0.001) with genome coverage (**Table 2**); that is machine learning models derived from CRE peak sequence data sets that covered a smaller portion of the genome were significantly more accurate. Correlations with either peak number (p=0.2637) or average peak width were non-significant (p=0.2365). The sequencing methods separated into roughly two categories. STARR-seq (90.4%), DNase-seq (90.4%), and UMI-STARR-seq (88.3%) and FAIRE-seq (86.2%) demonstrated the strongest sequence-activity association, while ATAC-seq (83.2%) and ChIP-seq (H3K4me1 – 77.8%, H3K4me3 – 82.2%, and H3K27ac – 80.5%) demonstrated the weakest associations. Our results suggest that STARR-seq and DNase-seq data sets demonstrate the strongest sequence-activity relationships of those evaluated here, and, consequently, are most appropriate for training machine learning models.

### Evaluation of sequencing data for enhancer activity models

H3K4me1 and H3K27ac histone modification ChIP-seq (Chen et al., 2018; Butt et al., 2022) and STARR-seq (Yáñez-Cuna et al., 2014; Yáñez-Cuna et al., 2012; de Almeida et al., 2022) data sets were used to train and evaluate sequence models for a binary prediction of enhancer activity. Experimental measurements of enhancer activity were used to determine if the observed disparity in model accuracies was strictly computational or inherent to the sequences themselves.

**Figure 3:**
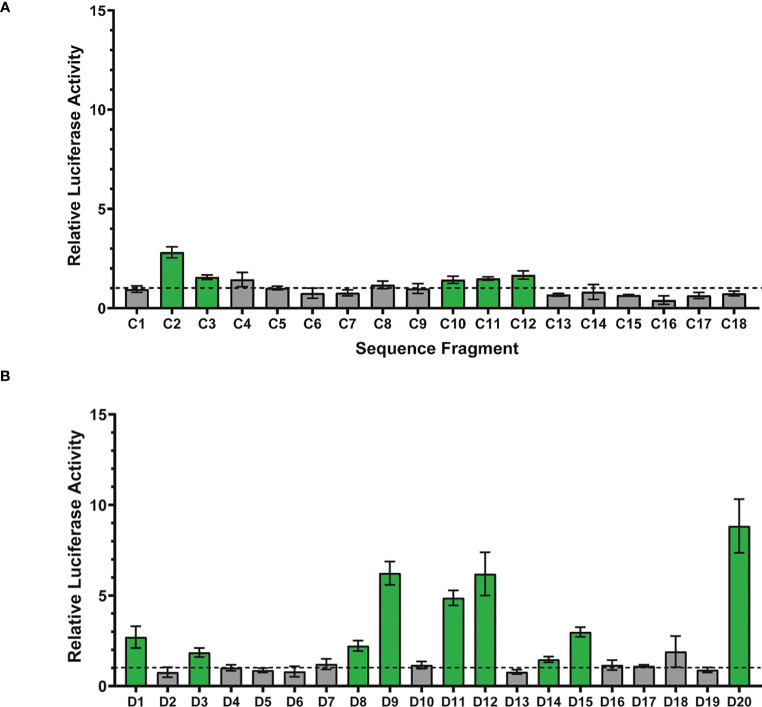
Assessment of enhancer activity detection using luciferase reporter assays. **(A)** Eighteen randomly-chosen H3K4me1 ChIP peaks were cloned and their enhancer activity quantitively measured using dual glo luciferase assays (6 replicates per fragment). Each bar represents the average relative luciferase activity for a given sequence fragment ± one standard deviation. Relative luciferase activity was computed as the normalized luciferase activity for the tested sequence fragment (firefly/renilla) divided by the normalized luciferase activity for a negative control sequence fragment (firefly/renilla). Statistical analysis to identify sequence fragments with enhancer activity significantly above 1 was performed using a one sample T-test with a p-value threshold of 0.0028 (0.05/18 statistical tests). ChIP-seq sequence fragments with enhancer activity significantly above 1 are shown in green and fragments with activity indistinguishable from 1 are depicted in gray. The horizontal dotted line represents relative luciferase activity of 1, equal to that of the negative control. **(B)** Sequences from twenty randomly-chosen DNase peaks were cloned and their enhancer activity quantitively measured using dual glo luciferase assays (6 replicates per fragment). Statistical analysis to identify sequence fragments with enhancer activity significantly above 1 was performed using a one sample T-test with a p-value threshold of 0.0025 (0.05/20 statistical tests).

Of the 18 H3K4me1 ChIP-seq sequence fragments tested for luciferase activity, 5 (28%) displayed activity significantly above 1 (**Figure 3A**). For these 5 active fragments, the relative luciferase activity averaged 1.8 (range 1.43-2.81); thus, while active above background, their relative activity is quite low. Substantially fewer H3K4me1 ChIP fragments were active than compared with the 81% (62 of 77) of STARR sequences reported in Arnold et al. (2013). This 28% enhancer activity rate is consistent, however, with the activity rate (26%) observed by Kwasnieski et al. (2014) when testing 2,100 regions identified by ChIP-seq using *cis*-regulatory element sequencing (CRE-seq).

Dogan et al. (2015) similarly observed high false-positive rates from H3K4me1 and H3K27ac histone modifications and found that occupancy by the TAL1, GATA1, SMAD1, and EP300 transcription factors were more accurate indicators of enhancer activity. ChIP-seq identified 4,915 DNA fragments bound by TAL1 in mouse G1E-ER4 cells. Thirty-nine (59%) of seventy randomly-chosen TAL1-occupied DNA fragments demonstrated enhancer activity in human K562 cells when tested in luciferase reporter assays. Comparisons across species (mouse to human) may have affected detection of enhancer activity rates; in future work, it would be interesting to repeat the analyses using *Drosophila* S2 cells to enable direct comparison.

**Figure 4:**
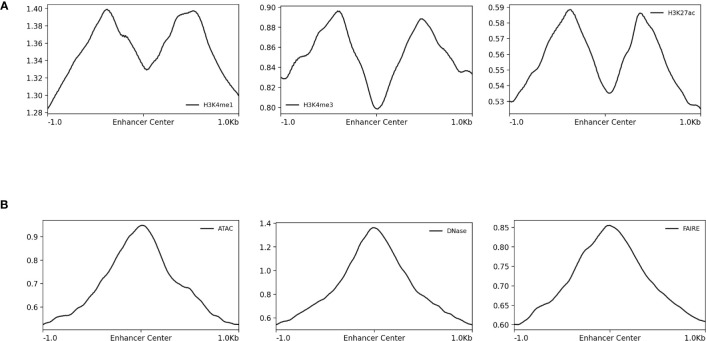
Normalized read-depth profiles around STARR-seq peak centers. Normalized read-depth profiles for the **(A)** H3K4me1, H3K4me3, and H3k27ac ChIP-seq and **(B)** ATAC-seq, DNase-seq, and FAIRE-seq data sets calculated around the centers of the STARR-seq peaks. Notably, the summits of the ATAC-seq, DNase-seq, and FAIRE-seq peaks were centered on the STARR-seq peaks, which was not true for the H3K4me1, H3K4me3, and H3k27ac ChIP-seq peaks.

Histones mark the boundaries of enhancers in regions of open chromatin (Shlyueva et al., 2014). ChIP-seq peaks are most accurately interpreted as marking those bounding histones rather than the enhancers sequences themselves which may be offset from the ChIP-seq peak centers. This is made clearer when sequence read depth is examined for H3K4me1, H3K4me3, and H3K27ac data and shown alongside STARR-seq data (**Figure 4A**). By utilizing windows centered on the ChIP-seq peaks, only part of the enhancer most proximal to bound histones is captured and depending on the enhancer length, the enhancer and TFBS within it may not be captured at all. More sophisticated approaches such as those proposed by Sethi et al. (2020) that match shapes of corresponding pairs of peaks to accurately localize the enhancer region from ChIP-seq data are needed to extract high-quality enhancer sequences for machine learning. In the absence of these more sophisticated analytical approaches, conclusions related to enhancers based on histone modification ChIP-seq data should be interpreted with caution since they may be capturing sequence patterns associated with the histone binding rather than sequences underlying enhancer function.

### Evaluation of sequencing data for regulatory activity models

Chromatin accessibility assays such as DNase-seq and FAIRE-seq are used to identify a broad range of regulatory elements and infer the specificity of their activity across experimental conditions such as different tissues and developmental stages (Song et al., 2011; Davie et al., 2015; Murtha et al., 2015; Pearson et al., 2016). Along with STARR-seq, the DNase-seq model produced the most accurate predictions, suggesting that DNase-seq is likely better suited than FAIRE-seq or ATAC-seq for training models to predict regulatory activity from sequence alone.

Of the 20 DNase-seq sequence fragments tested for luciferase activity, 9 (45%) display activity significantly above 1 (see **Figure 3B**). For these 9 active fragments, the relative luciferase activity averaged 4.2 (range 1.47-8.84). Luciferase activity for the 9 DNA-seq peak sequences displaying activity significantly above one is significantly higher than the luciferase activity for the 5 ChIP-seq peak sequences (Mann-Whitney, p=0.04). A greater fraction of DNase fragments were active than the H3K4me1 fragments tested (28%) and substantially lower than the observed activity level of STARR sequences (81%, 62 of 77) tested by Arnold et al. (2013). For fragments with enhancer activity, that activity was higher for the DNase-associated enhancers than the H3K4me1-associated enhancers, consistent with previous observations of unexpected depletion of H3K4me1 in highly-active enhancers (Henriques et al., 2018).

The differences in the luciferase assay activities compared with the similarly high prediction accuracies for the sequence models confirm that the patterns found by the machine learning models are specific to the sequencing assay used to generate the training data. DNase-trained models are more appropriate for identifying the larger set of regularity elements, while STARR-trained models should be prioritized if predicting enhancer activity is the primary goal. Given the differences in indirect and direct methods for identifying enhancers, it is maybe not surprising that an assay that directly queries the enhancing properties of underlying nucleotide sequence would be best suited for training of machine learning models. Yet, there are limitations to these direct methods including the fact that they test DNA sequences in the absence of chromatin context. For example, a sequence that displays high enhancer activity but is rarely found in open chromatin may not strongly influence gene expression *in vivo*. If the goal is to identify enhancers that are active in a particular biological context, STARR-trained models could be combined with tissue-, life stage-, and environmental-specific DNase-trained models.

### Application of enhancer sequence models to other sequence methods

The STARR-seq sequence model was applied to peak sequences from the other sequencing methods to estimate the fraction of overall peaks with enhancer activity. For each sequencing technique, 500-bp sequences centered at the peak summits were extracted. The model was trained on the STARR-seq sequences and an equal number of randomly-selected 500-bp control sequences. The fraction of peak window sequences predicted as enhancers are given in **Table 3**. From as few as 26.9% (H3K4me3 ChIP-seq) to as many as 60.1% (DNase-seq) of peak window sequences were predicted to have enhancer activity. The sequence activity counts from the DNase-seq and H3K4me1 ChIP-seq luciferase assays were compared with the computational sequence predictions from the STARR-seq sequence model using Binomial tests. No significance differences in the fraction of active sequences were observed for either H3K4me1 ChIP-seq (k=5, n=18, p=0.354, p-value=0.626) or DNase-seq (k=9, n=20, p=0.601, p-value=0.178). These results suggest that STARR-seq sequence models may be useful for estimating the fraction of and even filtering peaks from other sequencing methods for enhancer activity. To address this question in greater depth, however, future work should perform a similar analysis with larger sample sizes.

**Table 3:** STARR sequence model enhancer predictions and UMI-STARR-seq overlaps.

Data Set	Predicted Enhancers (%)	Positive Prediction and Overlap*	Positive Prediction and No Overlap*	Negative Prediction and Overlap*	Negative Prediction and No Overlap*
ATAC-seq	39.3%	15.3%	24.0%	12.7%	47.9%
DNase-seq	60.1%	42.6%	17.5%	19.0%	20.8%
FAIRE-seq	40.6%	18.9%	21.7%	16.3%	43.0%
H3K27ac ChIP-seq	26.9%	10.8%	16.2%	12.9%	60.1%
H3K4me1 ChIP-seq	35.4%	12.2%	23.3%	5.9%	58.6%
H3K4me3 ChIP-seq	26.5%	9.8%	17.0%	10.8%	62.4%

* The STARR-seq sequence model was applied to other sequencing method to assess the potential for finding additional enhancers not captured by STARR-seq. UMI-STARR-seq is more a sensitive method than STARR-seq so validation was performed by comparing overlaps of positive predictions with the UMI-STARR-seq peaks. Positive prediction means that the sequence fragment was predicted to be an enhancer by the STARR-seq sequence model. Overlap means the peak was overlapped by a UMI-STARR-seq peak.

UMI-STARR-seq is a more sensitive method than STARR-seq, identifying more enhancers under the same experimental conditions. Predictions from the STARR-seq sequence models were compared with overlaps with the UMI-STARR-seq peaks to assess the potential to identify additional enhancers by applying the STARR-seq sequence models to peaks from other sequencing methods (see **Table 3**). DNase-seq (42.6%) had the highest fraction of peaks with positive predictions that overlap UMI-STARR-seq peaks. The other sequencing methods had fewer than half as many overlapping peaks (9.8 – 18.9%). Applying STARR-seq sequence models to other sequencing techniques appears to be a promising approach for augmenting the enhancers found by STARR-seq alone.

## Conclusions

Available methods for computation identification of enhancers and other CREs are increasing in number, reliability, and accuracy. For example, support vector machines (SVMs) using k-mer counts (Lee et al., 2011; Ghandi et al., 2014; Lee, 2016; Chen et al., 2018), Hidden Markov Models (HMMs, Schember and Halfon, 2021), and convolutional neural networks (CNNs) (Chen et al., 2018; Koo and Eddy, 2019; de Almeida et al., 2022; Rada-Iglesias, 2018) have demonstrated success in distinguishing enhancers from randomly-sampled sequences, even across species (Chen et al., 2018; Kelley, 2020; Schember and Halfon, 2021). In a few cases, sequence models have applied to *de novo* genome-wide computational prediction of enhancers (Kazemian et al., 2011; Lai et al., 2018; Schember and Halfon, 2021; de Almeida et al., 2022).

Establishing machine learning models as reliable methods for CRE prediction based on DNA sequence alone will require careful planning and extensive experimental validation. Critically, as demonstrated here, the choice of next generation sequencing assay data used to generate training data determines the capabilities of the resulting model. A range of ChIP-seq (histone modification and transcription factor binding), chromatin accessibility, and STARR-seq data sets have been used in computational modeling of enhancers and other regulatory elements. The choice of data is due, at least in part, to availability. ChIP-seq was one of the first widely-available, genome-wide methods for the identification of CREs. Using histone modification antibodies requires fewer experiments necessary compared with using a separate antibody for the binding of each transcription factor, resulting in lower costs and labor. More recently, ATAC-seq is favored over other chromatin accessibility assays since it requires less raw DNA as input material (as compared to DNase-seq) and offers a superior signal-to-noise ratio (as compared to FAIRE-seq). Yet, despite the experimental convenience of these sequencing methods, their strengths and limitations for training machine learning models are not yet widely appreciated.

Multiple factors that impact model accuracy must be considered. Enhancers and other regulatory elements constitute less than 5% the *D. melanogaster* genome according to the STARR-seq and chromatin accessibility data used in this study. False positive rates will be amplified by the imbalance of the noncoding to the rest of the genome and need to be tightly controlled to avoid overwhelming true positive predictions. Model performance depends substantially on the choice of input data; the (UMI) STARR-seq and DNase-seq data sets produced the most accurate models and should likely be preferred as training data for models of enhancer activity. All three of these next generation sequencing based methods show CREs covering in a small fraction of the *D. melanogaster* genome and have moderate peak sizes (>300bp). As observed above, sequencing methods with similar genome coverage, but smaller average peak width or with greater genome coverage result in less accurate models.

Mutagenesis assays have demonstrated that deletion of sequence at the ends of enhancers can substantially impact activity (Nardini et al., 2019). Modified histones bind at the boundaries of enhancers, which is observed in the location of the ChIP-seq peaks relative to the STARR-seq peak centers (**Figure 3**). Sequence analyses such as TF binding site motifs and reverse engineering the regulatory grammar that depend on ChIP-seq data need to appropriately account for the discrepancies in locations.

The differences in the type of data generated by the array of sequencing methods used to identify and characterize CREs and how these differences propagate into resulting machine learning models have yet to be sufficiently considered by the computational modeling community. Here, the sequence-activity relationships from ATAC-seq, DNase-seq, FAIRE-seq, H3K4me1, H3K4me3, and H3K27ac ChIP-seq, STARR-seq, and UMI-STARR-seq assay data from *D. melanogaster* were evaluated using machine learning models. DNase-seq and STARR-seq demonstrated the strong associations. Experimental validation with luciferase assays indicated a high false-positive rates for detection of enhancers by H3K4me1 ChIP-seq and DNase-seq data. We conclude that STARR-seq data are best suited for training models to identify enhancer activity from sequences, while DNase-seq data are well suited for training models on the broader class of regulatory elements, including their context-specific behavior. Our results complement previous work from Kwasnieski et al. (2014) and Dogan et al. (2015) evaluating histone modification and TF-binding ChIP sequencing. For consistency with prior work, the ATAC-seq, DNase-seq, and FAIRE-seq data presented here were processed using parameters for MACS from the original papers (Arnold et al., 2013; Davie et al., 2015). The ENCODE project (ENCODE Project Consortium, 2012) has since curated a newer set of best practices for processing these data as implemented by the ENCODE Uniform Data Processing Pipeline (Lee et al., 2016). According to the read-depth profiles in **Figure 4B**, the ATAC-seq, DNase-seq, and FAIRE-seq peak summits align well with the STARR-seq summits. Since we use the 500 bp windows centered on the peak summits, we do not believe our analyses are negatively impacted. Nonetheless, future work should validate the impact of processing the ATAC-seq, DNase-seq, and FAIRE-seq data using the ENCODE-recommended best practices on the analyses performed here.

## Data availability statement

The original contributions presented in the study are included in the article/**Supplementary Material**. Further inquiries can be directed to the corresponding authors.

## Author contributions

Designed research: MR, RN. Performed research: RN, KN, JP, MR. Analyzed data: RN, KN, JP, MR. Wrote the paper: RN, MR. All authors contributed to the article and approved the submitted version.
