# Supplementary material for: Prediction accuracy of regulatory elements from sequence varies by functional sequencing technique
Source: Front Cell Infect Microbiol. 2023 Aug 2;13:1182567. doi: 10.3389/fcimb.2023.1182567 (PMC10433755; doi:10.3389/fcimb.2023.1182567)
Supplement: Supplementary file 1 [file Table_1.docx]

**Supplemental Materials**

Ronald J. Nowling, Kimani Njoya, John G. Peters, Michelle M. Riehle

Table S1. Primers used to amplify genomic fragments

| Primer Name | Primer Sequence (5’ – 3’) |
| --- | --- |
| D1_For | TGCATTCCTAAATTGGGTTGA |
| D1_Rev | CGAAAGAAGGGAAACAACAA |
| D2_For | TTGTTCCGCTGAAGAAAGA |
| D2_Rev | TTGCCATTGACGTGTGTTC |
| D3_For | TTAGCCTAAAACGAAGACCTAGTG |
| D3_Rev | GCCATTGTGCAGCAGAG |
| D4_For | TGAACTTCAAAGCACATGGAG |
| D4_Rev | CAGGCTTCTTTGCGCTTA |
| D5_For | TGTTTTACAAAACGCGCAAA |
| D5_Rev | TTTCTATAGTGCCGTATTTCTATGG |
| D6_For | CGCTGATAATCACGAATTTGA |
| D6_Rev | CTCGCCTTAAGCCGTATTT |
| D7_For | TGGGCTATTTGTGGACTTATTTT |
| D7_Rev | TAGCCAGCCCTCGTAGAAA |
| D8_For | CGTCCCATGTTGTCCATTC |
| D8_Rev | GGCGTTCTAGTTCGAAGAT |
| D9_For | TGTCCTAAATAGGGTGAAAAATGA |
| D9_Rev | GCCAGTGGAAAAGAGAGAA |
| D10_For | AACCCCCGAATTATCTGGAG |
| D10_Rev | GAGCGAGTGAGTCGAAGAG |
| D11_For | GAGTGAGTGGGTGGCAAGAT |
| D11_Rev | TCCCGATCGAGGATCAAT |
| D12_For | ATCGCCGTTCAAGTTCAAGT |
| D12_Rev | CGAGGTTTCGCACACTATG |
| D13_For | TTTCAAGTGAGTTGACGCATTA |
| D13_Rev | TGGTAAAGGGAAGAAGGGTAA |
| D14_For | CCTCCTTCTGGAAAATGCAA |
| D14_Rev | TCTCGAACGCAGGGATTAG |
| D15_For | TCGCATTCCATACTATTTTCGAT |
| D15_Rev | GTATTAAAGCCTGCCTGTGA |
| D16_For | CGGCGATTGTGCAAATATAA |
| D16_Rev | TCGATGCAATCGCCTATCT |
| D17_For | AAAAATTGCAAAAATTCGTAAAAGA |
| D17_Rev | CAGTACCCAACAGCGAGTT |
| D18_For | TTCCCCCGTTCCTTTGATA |
| D18_Rev | AAAGCTGCCAGTCCGATTA |
| D19_For | AATTAGTTAAAATACAATCCCATTGC |
| D19_Rev | ATTGATTTTTGTCATTGATGGT |
| D20_For | GCTACTGTGCATGAAATGCTTC |
| D20_Rev | ATAATAATACAAGAGAGCAGGGTACA |
| C1_For | TTGACCACCTCCTTCCAGTC |
| C1_Rev | GAACAAGAGCACGGCCTTA |
| C2_For | AACGACGGAAATGAAGTTGG |
| C2_Rev | GTTTTATTTGGGATACCTTTCAA |
| C3_For | AAATAAGGGCCGCAAGTTTT |
| C3_Rev | GTGTCGAATTTATGGCTGTTT |
| C4_For | CGTGGCTGGTTAATTGAAAA |
| C4_Rev | GACCCTCTTAGCGTTTCAC |
| C5_For | CTCCAAATTCACAAACCTTATCTT |
| C5_Rev | TGACGTCGCCTTACTCAAA |
| C6_For | GCTCAGCATCTCCAGCATTT |
| C6_Rev | GCTTGTTTTCCAACAGCAT |
| C7_For | GCATTTGACTTTATTTCCCTGA |
| C7_Rev | TACCCAACCCAACCATCAC |
| C8_For | TTCAACCAGGATTTGTACTCCTC |
| C8_Rev | ATCCTTGCTCACCTTCAGC |
| C9_For | TACTTTGCCCACTGGCAACT |
| C9_Rev | GAAATCTTTTTGTGTTATATTTTTCG |
| C10_For | TGCAATTAACACAACGCAAA |
| C10_Rev | GTTGATTTAGCTGAGGTAGTCG |
| C11_For | GCTTGTTTGAAAGGACTGTGC |
| C11_Rev | CTTGCGACGGAGTTAAAAG |
| C12_For | TCCGGGCTGTGTTACTTTTT |
| C12_Rev | GAAGAATGGGGAGTAGCAG |
| C13_For | AATTTGCACTTTGGCATTGA |
| C13_Rev | CCTCAGCTGGGATATTCG |
| C14_For | ACACGAGGGTAAACCGAATG |
| C14_Rev | CCATTTAATGTAAAATGCTAGGTT |
| C15_For | CGTTTAGGTGGGAGCCATTA |
| C15_Rev | GGGCAGATCGAATTACTCT |
| C16_For | TCTTGAGAAGATAAGGATAGAAACCAA |
| C16_Rev | CAGATCCACAGATCATCACC |
| C17_For | AAGACAAGTTTAAACAGCCTACGA |
| C17_Rev | AGAAAGGGGAGCCAGAACT |
| C18_For | AACGCCGACTTGCTTAAAAA |
| C18_Rev | TCCTAGCCAGCCTTAACGA |
| Positive Control ApE5_For | GAGCCCGGCTCTATTCACACTTT |
| Positive Control ApE6_Rev | TCGCCCTTCCAGGACTATGTTT |
| Negative Control 2L_For | TGAAAGTCGAGGAATTCAACC |
| Negative Control 2L_Rev | ATCGAGCTACGAATTTGGCTTT |

Table S2. Sequences of all tested fragments.

>D1

TGCATTCCTAAATTGGGTTGAGTTCTATGAAGTGTGGGCATTGTTCACACATGATACAAAGAAGAAAAGGCGAAAATTAAAGAACTGAGCAACTGATGACAGCTAATCTTGCTTTCTTATTATCTGAAAATGTGGGCATGTAAAATTCCATAATTTATGAATCATCCCATCCAACAACAATGCACAAATATAATTACTTATATGTACATTCATATGTACATATGTATGTGTATGTGAAACGTCACACAAATCTACTATATTCACATACAAATTTATATGTGAGTGTGGAATTTAAGCAAAACGGATTGCAAGAATAATCGCAATGTTAATTGCAAAATGCGAGAAGTGAAAACAGAGTTGCCACGTCATACGGTGGCAGCCCTGATCGAGAGAAACTACGAAAGCGCTGACTAACTGTACTTCATCCCATTTTTCTTCTACTTTGCGAAGTTTTTTTTTATATTTCTCTTTTGTTGTTTCCCTTCTTTCG

>D2

TTGTTCCGCTGAAGAAAGAGAGTGAGGGAGAGAGGGAGCGTGCGAGAGAGTGCGGTTTCTTATCACTCTCTGCGAATACGTAGCCCTACGTTACGTAGCGCCCGCTACGTTAGTGCGAGGAAATGAGAGCGCGATAATCAGGGCGTCGAAATATCAACAAGTATATCGAGTTTTGGCTTCCACGTCGGCGGCACACGCGATTTCCAGCCTTCCAGCTGTATATGTGTACATTATTCTAATTTTGATTAGTTGTTCTGCTTAACCCGCCTCGCAACAGTAACGAATCGACGATTCCACCACGTGGTGAGTTCGGTAACACGTGCCGTTCCAGCT

>D3

TTAGCCTAAAACGAAGACCTAGTGTTAGGCTTTCTAATATCCCAACTTATAAATATTATTTACATACATATATACATATGTACATTGTATTTTCATATGTTCAGTGGAGAGATCGAGAGATGCGCTTCTCAAATTCCATTTTTTCCGTTATACCCCGTCTTTCACTGTCCGCCTCCACTATCTCTATCTCTCCGCATTTTCTCTTCTCTGTCCGCCGCACACACTCTCGTTGGCGTCTGTCTGCTGTCATCAGTTGCTGATTACTTGTTCACCAAACCGCAGTTTAGCGCTGGCGATGCCGGCGGCAGCAGACGTAACGACGACGACGGTCGCGTCTGATAGACAGCTGTCTCCAATTGGAATGTTATCTCCGGTTACGTCAGTCAAAATGACGCATCGTGTTTTGGTAACAGGAAAGCATAAAAGCGAGAAGAGCACCAATGTGCGGCACTCTGCTGCACAATGGC

>D4

TGAACTTCAAAGCACATGGAGCGATTTAGAGTAGAAGGGAAGAGGAGGGGTTAATAAACAAGCCACCAAAAGGTAAATAAATAAGCACAAAATGCCGAAAATAGCGCACAAGGGATAAACCAGATGGAAAGAGCGAGAGGAAGAGAGAGAGAAGGAGCAGCTGCTGCGCCGACGCCTGCTGCTCTTGAGCTTCTTCTTTCCATCTCCCTTCCCCAACCCCATTCACTCGTCATCCAATGGCTCTCCACAAATTGGAAGCAATTACAATAGCAGCAACAACAAATGCTCTTTGACTTTTTACAGCAGCCGGAGCAATAACTAAATACGGTAGACCGTCGATAAAGTGAACCAAACACAATTTAGTGGTTGCAAATAAATTTTTAGAACCAAATTATCATAAAGCTTGAAATGTTTTTTTTTTCAAATTTACTCCAATTGGGTATTTTTTTAAGCGCAAAGAAGCCTG

>D5

TGTTTTACAAAACGCGCAAAAATCTTTAAATAGTGTACATAATGTATGCATATAGGTACATATTGAGAACTTTACCTTTTATATATGTATGTACATATAATCCACAAATTTACAGAAGTATCTTTACCATACCCAATAATACTCTATATACACGAAATATTCTAATCATAAATGGACGTTTGCCGCGATGACGAAGACTTTGCCTATTGTAGATTTTTATTTGCCCATGGTTTGTGTTGCTTTTGTTGTGGGCACTTCAAAAGTCGTGTGCGCGTTGTTGTTGTGTTGTGGTGTTTGGTTGTTGTATATCGGTTGTTTACTTTTACGGTAAATGCGAAAAGCAACAACACTAAGGACCACAAAAAAGCGCGCGCTCGTCGCGCGTATCTAGATAAATATCTGTGAAAATACACTTGAGGCCAAGATACTGCACAGCGGGCGCCATAGAAATACGGCACTATAGAAA

>D6

CGCTGATAATCACGAATTTGACATTGCTACCACATTCGGTGCGTGGACTCTGAAAGCTCTGAGTGTTTTGTTTATGCAAAGCTTTTTTGGGCTATCGCGTGGTAAGTAGCCGAAAGAGAAAGCTCTCTTATACGGAAGATGAAGAGTGTGATTCATGAAAATGTATAAGAACGCGGGTCCAAAAAGTCAAGGGAGTTCTAGTGAAATGAAAAGTTCCAAAGGTTTTGAAATCGTTTTATTTTCTCGTTCGTATAATTATTGGGTGTCGATCTTTGTTGGGCAGTGTAAAGCACAAACTTTGAGCTTCATCATACATATCATATGTAAAGCCGGGACGAAAGCTTATGATTCTGTTAAGTGTCCGCCCAAGATAACATTTCTCCAGCCCTTCAAAT

CTTCAAATAAATACGGCTTAAGGCGAG

>D7

TGGGCTATTTGTGGACTTATTTTTAAAATACTAGATTGAACACGTTTCATATGGCAACCAAAAAAGGTTTTGTTTTGTTTTTTACAAGATTGTAAATACTTAATTTTTACAACTCAAAATTATAAATAGTGCCAGTGTTGGGTAATGTCAACTACGGTCACACTAATCTAAAACAATTTGAAGCAAACGGTATATTACACTAAGTTCAAGTTTTGAAGCAATGGGGCTTACACCAAACAAATATATAAATAATAATAACTTTCCGCCGAATATAAGCGTTCGCATCTTTTATTTCGATATATCTTAGACTTAGCTTAAGCCTATCGAATACTTATTCGATATTGCCTCGGAAGATCTATCGCAAGCGTGAGGCCCACCTCTAGTTGGTGTGCGCGGAGTGGAGTGAAATAAATCGAAGTAAATTGCGTCGAGCGCTGCAAAATAATGGATCGCACTTTCTACGAGGGCTGGCTA

>D8

CGTCCCATGTTGTCCATTCGGTCCCACCGTGCACACAACTCAGCACAGCACGGCACAGCAGCAACAAAAGCGCCGCAAAAGGGAACTGGTATTATGGCATCCCGCTCGCACTTGCACTCCAAGCACGCACACATGCAAGCGCAGACACCTCGCGACACGATTGTCGTCAGTTTGTCTGCAATGCTCAGCTTCAGCGCCATGCGCTCTCTCTCTGTTGCTCGCTCTCTAGCTCCCCCTCTTACTCTCTCTCCCACTGCTGCACGCACACGAACACCCACACGAACAGCAGCAACAACAACAGCAGCGCACAGCAGACAGGCAGGCACTTTACGCCTGTGTGCGTGAGCATTTCAGAGTGAACCGCAGTCGACGGCGGCGCCGGCAGCGACGCCGGCCGAGGCGAGGCAGTCTAGAACGTATCTTCGAACTAGAACGCC

>D9

TGTCCTAAATAGGGTGAAAAATGAAATGCGGGGGAGAAATTGTGTTAAAATAATAATTTATGTGCCTCTTTGGGCACTGTGTTTTGAATTTCTGTATTTCTTTTCTTTTGCTTTTTGCTGTGCTCATTTCGGTTTTTGGTTTTTCTCTTTTTTTTTGTCAACATATGTGTAGTTTGTTGTTCATCTTGACTACTTTTTTACATTTTTTTTGCGTTGTTATTTTTTCTTTGTCTTTTTTTCGCTTTTGTCATGCGCTCGCTTCGTTCGTATATTTTCGCACGTACGCTTGTACTTGTTGCTGTTGTTGTTGTTTGGGGTCACGTCATGTGCTGGTTCTCTCTCGCCCCGTCTCTTTTGCGTTAGTCATAGTATGGTCCGCCAAATAAGAGAGAGAGTTCCACGTGAGCCGTTTCTCTTTGAGCT

>D10

AACCCCCGAATTATCTGGAGAACTCTCCCCCCCCCCCCCCCCCTTACCACTGCCATTCCTCATCTCTGTTTTTTTTGTGTGACTGCCTCGTCGTTGGCCATTGTTTTTTACTCTTGCAATCAGAAAAAACTTGATCTGATGTTCGGTTTGGTTTCTCGTTTACCTCCACACCACTCCCCATAAACGTGTCCGCCTTATCATATGTTCCCCGTTGTCTGTGTCTGTGCATTGCCAAATCTTTCGCCGACTTTTTGTTTTCATTATTTTCGCAAAGTGCTGGGAGAGCCGCCCGTGGAAAGAAGAAACCAGTTCCACCAGATGCCGGCCGGCACACAAACGGCGAAACACAGACGGCGGCACACATTCGCCTCGGCCTGTTCACTTTTTGCACTTTTTCGAGCGGTTGCATGGTTAACTCCCCGTAAAGCACATACCCTCCGATCCCGACTCTCGATCGCCGGAACACACGACGGCTCTTCGACTCACTCGCTC

>D11

GAGTGAGTGGGTGGCAAGATAGCGGGCGGCGGGCAGGAAGTGAAACGAATGCTAACCCAAGCCAGCAAAAAAAAAAAAACAAATCAGTGGTTCCGATTCGCAGACGTGTGTGCATGTGATTCAGAGCAAACAAAACGTACGAAATGCAAAACATGTGGATCATTTTCCTCATCATCGGATTGCTGGTGCTCGGGCTGCTGGTTCTGCTAATTATTGCGGCGCGGTATCAGCGAGATTATTGGCGCTATCTGGACATTCCACACGAGAGACCCAAAAAGCTATGGCCTATCATTAGGCAAATAATGACGCAGACATTGAGTACGGAGGCTATGAAGGCGGAGCACTACTCGGCCATATACAAGAAGTTCAAGGGAAGCGGTCCCTTCTGCGGATTCTACGCCCTGTTGCAGCCACGCGCATTGATCCTCGATCGGGA

>D12

ATCGCCGTTCAAGTTCAAGTTCTCAAGTTCTGTTTGCAGATATCAAATTGTGGTTTATGAGAAACATTTGTCGGGCAGCCCAGTTCCTGCCCAGACCCCCAGTCCCGTTTCCAAAGTTCTGATGGGGAGTGTTGTATATCCGCTTGTGTATCCAATAACCGCAGCGCAGCCCCCAAATGCTTTTGTTATTTATTATCGGGCTTTTGGACTCGGGTGCACGGATAATGATGATGACTCAATTGACGTACAATGGCCAACTTTCAAGTGAAGGAATTAAAGGCAAAGTATCATATCAAGTTCTCCCCATTCATATTCATGTTGGGACCCCGGCAAATCAGCTGATCTCGGCAGTTTCTCAACTCACGGCATATGGAGTAGAGTATGACAAATGTATCGCATATCGCTATATCGCTCCACGGTCATAGTGTGCGAAACCTCG

>D13

TTTCAAGTGAGTTGACGCATTAGCAGCTGAGTTACTTGAGTTGGTTGGAGTACTCGGTTTGTTTGAAGGCATGCCGCTCACGTGCCCCGATAAGAAATGTTCATAGTTGTAGGGATCGCAGTAGGGCTTTCGCATGTATTACCTCCCACAGTTTTATCTACTGAACCTCTTTAATCTTAACTGATTATTTAGTTTTTTACCGGTTCTTGTAGCGTTTGTTTTGCTTGGCCATACTCTAGATATAGATTTATTTTTAGCTGAAGTCCTCATATACACGAATATAAGTTATACGAATTTTCGGTATTGTATATTTATCATTCCCAAAAATAAAATACTCTCAACAAATATGATGAAAATGCAGCGCGTGCAGCAAATGAAGCACGGCAAATTGCTTTAATTCAATCAATTGCTCTCGCCTCTGTATAAATATAATATATAGATATTACCCTTCTTCCCTTTACCA

>D14

CCTCCTTCTGGAAAATGCAAAATTTGCATTCTATTGTGAAATGTGGCTGCATGCGGTTGCCAAAAATAATTTTGGTGACATTGCACTTCTATAAATAGGAAAATCGATTCATTTCCCTATACATTTGTATGGGAACCCCTTTTCGCCTTTTTCATTGTCACGTGTCTTTCATTTCAGCTTTTCTTGTGTTTTGATTGCAATGCGCTTTGTTTTGTTGAAATTAGGTGGTAAACAAATGGGAAATATGATAAACATGTTTACCTACTTGCTGTTTAGTTATAAATGAAGGGTTATATAGCATATATATTCTATTATATGCTATACACATACAGATGTACATATTCATGGTTAAACGAGCGATTAGTCATAGATCTTATTAATGGTCTTATGAAATATTTGCCGTGGAAACGTAATTTTGAAAAGTTAAGAACTGTTTAAAAGGTTCCCTAAATTACCACCCAAAAAAAaTATTATGAACTAATCCCTGCGTTCGAGA

>D15

TCGCATTCCATACTATTTTCGATGCTGTTGCTGCCCGGCATCTGTTGCTGGCCTATGCCTTGATTGTTCCAATTTTGGCTATGGCCAATGATGGCAAGTCATTGATAAAAATAGAGTAGATCATCCCCTGGATTCCCCCTTTTCTGAAACTGCTCTCTTTGTGTCTCTGCCCGAAGATATCAATGGGGAGAAATCCATCAGACTGCCATTTTGGGGTGTTATTTTGAACGGGGGAATGGGGGGCGAAAATGGAAATTTATCTGCTAATAGCACATCTGTTTGTCACATGAATAAAGTTGGGGGTACCGACTGGGTGACATGAAGTCATTTATGTCACACGTGTGCATTGTTTCTGAACCAAGTTTGGCCTTAGTTGGGTACAACTGATAGCAATCAAACAATTTTTAATAGATCTAGTGGCTCACAGGCAGGCTTTAATAC

>D16

CGGCGATTGTGCAAATATAAGATAGTAAACCACAAATTTGGAGTATTATAAGAAATATATAACGAATTTTAAGAAAATAATAGTTGTTATGGATCTTACTTGTGTGCTACTCATTAATAATAGAATTTTGTGCTTTTATTTCATTTTTCAACTATTTGATATATTTTTGAACTGCCTAGATTTCATACTTTGAAATTCGCACACTCGATTACGATGAGTCGATATCTTTAAAAATCGATAGTACCATGTGCAACTATCGGTTGTCTGCCCATGAGCACTCATTTCGCCCATTCCTAAAAGAAATCATTCAATTTTGAAAGACTTGAATGAAACCATGAAAAATGGCCAAGAATAAGTCGACTGTGTACCTAGGATACGAG

>D17

AAAAATTGCAAAAATTCGTAAAAGACTAAAGTGGAAAGCAAAAATTCTAAAACCCTCGTAAAAATTGTAAATTTTGAAAAATCTTAATTTTTTAAACAAATGCTTTGTAATTTAGTTTTAACAATTAGCTGCAACCAGCCGACATTTTGGTTTCTCACACATACACATGCCCATGCGCACAGACACATGCAAGCAACCACCACTTTGCCTTGACTTGTGCGCGAGGGAACGAGACAAGCATACGTTGTTGAAGCTATCGCACCGTTTATCGCCCAATCGATAGGAATACGTTAAATTTTGATTTTGAATTCGCAGGAACGAAAGTACAAGCACATTGGCGCCAATCACTTGCTTGAAATATGCAGTCGTCTGGGTCCACTGGTGGATCGCGAGCT

TCCGCCCAGTGTTCCGGGCATCAACTCGCTGTTGGGTACTG

>D18

TTCCCCCGTTCCTTTGATAAAAGTTTGCTCCCCTCACATCTGGGCAAACTTTCTCGATCATAACTCATAATATTCCCGGTTTTCGAGTGAATGTGATTCAGAAATGTGGATGATGGGCTGCCGGCGTAGCTTATTAAGTAGCACGTCAGCGAGCGCATAAATTCCGGTCAGCAGATACAACAGATATCAGATACAGTTTGGGGACTGACTCTTTCGGGGATCCGTCCGTATACATATTCTATTTTCATGTTCGCCATTGCGACGGACCAGACCCTGAGATTTTGATGAATGTGCGCGTCGCCAAAGAAAAATGTACGAAAGCGCTGCTCATGCATGAGAATTTTACGAGAAAATAATTGCGGAACGATATACGCGCCGATATTGTGGGCTTAATATTAATTGGCGGTTAGTGGAGAAAACTTACCAGATAATCGGACTGGCAGCTTT

>D19

AATTAGTTAAAATACAATCCCATTGCGGAAAATATTTAATTTTTTACGATAACAAAACTTCTCAAAGTGGAATCTTTTCTAATGATGTGCCTGAACATGCTGTGTCAAATGTATATCGCAAGAAATGTAATTTCAATGTTAGTGTTATACAGTACGTATACGTATACCAATGTTGTGTCAAATATTGAATACATTTAAGATGTAGTTCCAATGTAAGAAATACTGTAGACCTTTATTAGATTGAGCTATTAAGTATGTAAACCAATTTATCAGTTATTCCGAATAATTATTAACTCGCGTAGCATAGACTTTCACGTCTGTCCGCCATTTTTTCATCAGGGACGATCGCAAGTGGAACATGTAAACACCGTTTGGAATTGGAATCTCCTTTATTGTGTTTTCATCAACAGAAAGCCGTTGAAAGTTATATTTTGCTAAAGGTTATACCATCAATGACAAAAATCAAT

>D20

GCTACTGTGCATGAAATGCTTCATTCATAAAAAATAAAAAATTAAGGCCAAAAAAAAAGAAAATAAAAAATAAAAACACCCCGAAGCCCGCAGTCCCCCGAAGACCCCCTTTATTGAACGCCCCCAGTGTGTCGCCTTCATTATATGCAAATTTCAATTATTTGCACAAATCAGACGCTCAAGATCTGCCAGATCCAGCCACGACCAGGCATTATTATCAATCCCCCGCAAAAAAGGCTAAAGCTAAAGCTAAAGCCCAAAGAAGAGAGCGAAAAATATTGTTCAATTTCGTTCTTCGCTTTTTTTTTATCGGAGCAAACAGGAAGAGCCATAATATAATTTATTGTATTAATGCTGTCCCGCATAACGTCAGCATCATCATCATCAGCAACATCATTATCGCCATCAGAGTTCGTTCAATGGGCAGCATTTCATTGATACCCTTCTTATATTATAAATACGTATATATGTACCCTGCTCTCTTGTATTATTAT

>C1

TTGACCACCTCCTTCCAGTCGCTATACGACTGAATTTCGAACTCCAGCTCCTGAATGCGATCGTTGGCCACTTTCAAGCTTTGCGTTTGCTTCTCGTGATTGGCGCGCAGCTGCTCGAATCCGGAAGCCCTGGCCTGTAACTCCTCATTGAGTTGACGTAGGCGCTCCAGTTCGTTGCGCGTAGACTGCAGTTCGAGATCGGCCCGCTGGGCTATGCGGCGATATTCGCTCAGTTCGTTGTTGATGCACAGGTGCACATCGCGGGCATCCTCCTTCTGCAGGTACTTCTCCTTCTGCAACTTGAGCGTGGATTCATCGAACTTAAGCCTAAGTTGGTTCAACTCGGCGGTGGCCCGACTGGCCTCATTCTTCAAGACCAGCTCTCGCTTACGAAGAGCCTTAAGATCGTCCTGCAGCTCCAAATTCTTCTTGCTGGTGATGTCGCACTGATCCTTTAAGGCCGTGCTCTTGTTC

>C2

AACGACGGAAATGAAGTTGGTTAAGAAGTGCTCCACCTACTTGGTGATATGCCTGGGTAAAGTTCTGCATGCTCGTTCGAATCTGCCGAGTTTTGAGTGCCAACTAGAGATACGCAGCACACCGGCGAGATACTTTTTGGTGCATGTGTGTGGGCGGAGTATTGTGCATGTGTGAGACTTTAACGCATTATGATGCACTGATGCATCCGTATGATTCCATAGGTGTGGGGTTTTAATAACCCAACTTCAGCAATTGGAGACTCTAACGATGAGTAACTGGGCCAAGTGTGCGTCACACAATCAGGCACAAGAAAACTCTACAAAAATGTGTGCTTATCAGTGCAATTCAGATAAGCACTGTGTTTACTCATTGTTGCCCCATCACTTGGTGTGCTTAACTTTAGGGCGTGGTGAATCAGTGAGTCCAGAGGGAAATCTTTTTGGATTGAAAGGTATCCCAAATAAAAC

>C3

AAATAAGGGCCGCAAGTTTTCCAATACTTTCATTCATACGCAGAATTCCATATGGCTTCCATGGAATGTTTGCTTATCTGAAATGTATTCCTGATAGCGATTCGCGTTCAGATGCCTTGAAATCCGACATCCGAAATCCTATCCGGCATTCCATTCACCCACATTTGCGGATAATGTTTATGCACTCGCCGAATGAAATTCAATACTCTGCGTTGAGTTCATGAGTAATGCAACCCAGTAATGCAGCCCACCTTGACTTAATTTGCCGCGTCTCTTTTTACTTTGAAAGTTGCAACGAAAACAGAATTCACACGGAACCGACTTTCAGCGGCGCTTGGCTTTTTCCATTCAATGTCAGGCCGAAAGGTGAACTCTGGCCAGAACCGCAATCAAAGGCAATCCCACCATAAATCACTAATGACACCAACTTTATCTGTCTGTTTAAACAGCCATAAATTCGACAC

>C4

CGTGGCTGGTTAATTGAAAAGATATATGCCGCATTTCCGCATCTTGCATCTCGAATCTCGCTGCCCAGCGAAAACACTAGCCAGCTGGATGGATGGATAGCTAGATAGATAGATGGATAGATAGATAGATAGATATATGGATGAATGCACGATCGCCGGGGCTGAGAACGAATGCAATTAATGTCTAATTGTGCGCAATTATTGTCAGTTGTCAGCTGTGTGATGTGCCGCATCAGAATGCGCTCGCACCGAATTGAAACGATTCAAGTCGAGTGGAGATCTTCAAGGCGTCCACGTGGCGCATACGTAATGGCATCCATCTGACATTACTCATACGCACTGAAGCGCGTGCGGCTCACTGCTACCAGATGCATTGATGATTAACCTTCGTTGCTCAGGCGCGTCATGCGATTCAAATGGAATGATTTAAAATGTAGTGAAACGCTAAGAGGGTC

>C5

CTCCAAATTCACAAACCTTATCTTATAGCTTTTTTGCTTATTTCGTTTTTTCAGTCGAACGGCCACACAAACGGAGCCGGCGGCGACGGCGACCTAGACGGAACTTTTCGTGACCATCCGCTGGCGCAGCACACCAAACTGACCACCTCGCTGACGGCGGCGGGCACCGACGACGAGGATATGATTGACATCACACCCCGCTCCAGTCCGGGCGATGTGATCGGCGGCGGCGGAGGAGGAGGAAGCAGCAGTGGCTGTCACCGGGAAACGGCCACCGAATCGGACACCGAACGAGGTTTCGGACACTCTGGCGGCAACGGCGGCGGCAATCACCACAATCAGCGGACAATGCACCAGCATGTGCCAACTCACAGTGCCTTCGGCGATCCTATGGACGGTGAGTTCGGAGAGATCCATTGCGTAAAACATCCAATGTCGCTGTCTGTGGTGTTAATCAAGATCACGAACATTTGAGTAAGGCGACGTCA

>C6

GCTCAGCATCTCCAGCATTTCGGCCCGGCTCCACAGGTTGCGTTCCGGCCGAGGGCTATAGCCGAGGGAATCGCTCTTCACCGAACTGCCGGCGGAGTAGGGTGGCGACATGTGCGCCTTAATGCGCATGGGCGTCAGCAGTCCGTCGACATCGTCCACCACGCCGTTGCCCGTGGAGGCCGTGTGCAGGAGGTGGTTGATGTCCTGGCCGCTCGCGTTGCTCTGGTTGCTCTGGATGTGACCTTGGCTCTGACGCATGTTGTGCTCCACTTTACTTAGCAGCCGCATCTTCGTGTGAGCGGCCACCGTCGTCGGCGGAGAGGAGGAGCTGTTGGTCGCTCCTGCAGCTCCGTGACTGCCAGACGACGTGGAGCTATCCAAGTAGCGAGGCATTAACATGGATGTGGCTCTCAGGGTGCACCCTTTGATCAAGGTCTTAAATTCCCGATGCTGTTGGAAAACAAGC

>C7

GCATTTGACTTTATTTCCCTGATAGCGATTTCAACTTAGCGAGCATCCACTGTACTCCAAAGATTTACGACTCCTTCAATGTGGTATTGCGTTTTCGCTTATTTTATAGTGATTTATATCCATGCAATATCTATTGAGGAATTTCGCTTATATTGCCAAATCAGCGATAAGCGCTCATGCATATATGCACCGACTATCGTAATTACCGTTAACGCCCACCTGACGTCATCGGCGGCGATAGCGGCACCGTTACCTTCTCTCCGCATCTTCTCTCTTCCGTAGATATGTAACTGTCCATGCATGTGTGTATGTATGTCTGCCATTTCAGCCAGCATCATAAATATGTATGTATGTATGTACACACATATAGCACCCACATGCGTACCACTTTCGTTGTGCGTTCCACATGCAAGCAGATAAGCTGCATAATAAACCGCATTACGAGTGGGTTGCAAATTGTGGACCTGTGGGTGATGGTTGGGTTGGGTA

>C8

TTCAACCAGGATTTGTACTCCTCCTCGGGCGTCAACTTGGCCGCCTCATCGAGTGCCTCGGGCAGCTCCCACTCGCCCTGCAGCCCCATCCTGCCGCCATCGGTGAGCGCAAATGCCGCTGCGGCAGTGGCAGCGGCGGCCCACAATAGTGCAGCGGCGGCAGTGGCGGTCGCAGCGAATCAGGCCAGCTCCTCCGGCGGAATCGGAGGCGGTGGACTCGGCGGCCTGGGAGGACTGGGCGGCGGACCGGCGAGCGGACTGCTTGGCAGCAATGTGGTGCCCGGGAGCAGTAGTGTCGGGAGCGTGGGGCTGGGAATGAGTCCGGTGCTGAGCGGAGCGGCGGGACACTCGCTGCACAGTTCGCACAGGACGCACGCACACTCCCTCGCCCACGCACACACGCACCCGCACTCGCACACACACACGCATACGCATCAAACCAAAGAAGAGGACCTCATCGTGCCGCGCAGCGAAGCTGAAGGTGAGCAAGGAT

>C9

TACTTTGCCCACTGGCAACTGTAATTCGCTTTAGTGAATTAACCAGCTTAATTAGTTTAATTCGAGAGTGAAGACTGGAGCGTGGAAATGGAAATGTAGAGATTCCCGGCCAGTGATTTGGCAGTTAGTCGAGGACATTGCATTCCTGTCTGTCTGGAGGCTTTCCCCCTTCGTCTGGTTTTCGGACTGGCAGCCATTTAGTCCGACTTGATGTCGATGTCGTCGTTGTGTTTAATTATTCAAAACCATGTCATGTCTCTGTATAATTTTATGCATTGGCTAAACCAAGCGCCATGATGAAACCGATGGTCATTACGACCAATGATGATGATGATGATGACGCCTACACAGAGGCGCAAGAGTTAGACAGCAGACAGCTGACACCAAAACTATTGGCTACACAGAGCGAAAAATATAACACAAAAAGATTTC

>C10

TGCAATTAACACAACGCAAATTTCTTGTTTGTCTATCGACTTGTCACTTGGCTGGTTTTACAAGTCAGCTTTAATTTTGGTACATTTGTGGAGTGAACTTTTTATATATTCAGGTTTTTTCTATCACTTTTCGGTAAGCATTAAATAATGGGGCTTTAAAAAGTGAAGCTCGACTCACCCTCGAAATCATTCTCTGTAAATTGTTGCGTAAAATCCTCGCCGCCAGTGCGCGGATCCTTCGGCGCCTCGTCATCGCCGCTGGAGGAGTTCATGGTCCAAGGTCTGAAAAGAAGAGTGGAATTTAATATATATATATTTTTTTTTTTCGAGATAATTAAGCAGTGGAAAGGGGTGATGAACAAACATTTATAATGTCTGTGCGCTCTTATAAAACTGGTTGAGTAAGCCCTTATTCCAGCTTTGCAACAGCTTCTTATCGCTCTTATTTGAACCTTCATTACGACTACCTCAGCTAAATCAAC

>C11

GCTTGTTTGAAAGGACTGTGCAAAAAAAAAAAAAAAAAAAATCAAATTAGTTGCATGAATGGTGTGCTGGACGGATTAAGGGGGCGTGGCAGGAGCAGCAGGAGCTGCAGGTCCTGCAGGCCCTGAGTCTTTGTGGATATATACGGACTTTTGTGATTCATGTTTGTGTGCTAATTGCCACAGCATGGGGTTGGGCAAAGTCGAGAGCTTATCAGAAATTCTTATCGCATTACTTACTTATGCCGTTTACTTGGCTATTTGTCATAATTATCCAGCAACTAGGAAGATTCATTTGCCAAATGTTCTCAGTATATATCTATATATCTCCCTTCTAGCCTCCACTGGGAGCATTGAAAATTGATGTCCTTCAGTATGCATCAGGTTCCCTTTAGGAC

CAACCCAACCAGTCACATGTGCGCTCCATCTCGAATCTCAACTTTTAACTCCGTCGCAAG

>C12

TCCGGGCTGTGTTACTTTTTCGTCCAGTGTAGCTAGCTGTAAAAGAAGGGTGGTTCACAAAATTCCCCCCTATTTCTGTTTGCTTTATTATTTTAAAAGCTCCGTTGATTTAGAGCCCCCCACAATCGCTTCTTGCACAATTTCCACCCACCCACAATACCACACACACACTCAAAAATACTGTGTGTGTGAACTTCCGCCGAGTCTTTTGCTCCACCCCGCCGCCCACCTCCTCTCCCGCTGCACACTCCCACTATTCCTATTCAAAGGCAAACCCATACCACTCCCATTTCCATTTGCCTCCTCCTTCTCCTCGTTTTCTTCCTCCCCTTTCCTTTCCCCTTTCGTTGAGTGCCACATAATGTTATTTTTGTTTTTTCTATTTGCCTCTAAAATGAATTGTAGTCTCCACACCCCCGGCCTCTTTCtTTTCCTTCTCTCTGCTACTCCCCATTCTTC

>C13

AATTTGCACTTTGGCATTGAGCACCGGAAAAACGTGACTAGTTTTTTGCCCAGCCAAACAGAGTTATTCTTTATAGTATATACCACTAACTAGCTAACTATATCAGACCGAAGCGTGATTTCTTGTGCTTATCGTCATTTGTTTATTTTTGGCCCACAGCTGAATTTAAATAACACGAATGGACCCGAGTGCAGGTCGGAATGAAATCGAGATTTTGTATTATTTACGATAAGCCATGGAGCTCAGATATGCGGCGAGAACTTTGCCTCGCAAAAGATGACTTAAGCAGTTATTTTTAAATGGCTTGAACAATTCATAAAAGGGGATCACGGGTGGTATACTTAATGCGGGTTTATGAGGTGAAAGATTATTTGAGAGAAGTTAGGAAGTTAGGGTCTCTACTAAATACTCTTACTTTTTATTTTGTAAGATGTAATGTCATTTAATTCGTATTAAGTATTAATCATTCGAATATCCCAGCTGAGG

>C14

ACACGAGGGTAAACCGAATGGGCGAATTTCTAGCTTGGATGGCATGGATTGCGAGGTGTGAGCATCAGCAGCACTTTGCCAACAGCCTCTCGTATTTATAATTGCTGCAAATTGCCGCTTAAAAACTTGTGTTCCTGCCGGGGCATCGGGCCAAACTACTCGAGCCACTTACGACGATGTCAGCGTCATCAGCATGGGATGGGAGTGCCAGGCACGTGCTTGAAATAGACTCCATCACTAAATATAAACCATTTGGCATTTGCTATGACCCAAAAATTCAGATCGAAGTGGTGCGAAGAGAAGACCAAATCATAGGTGATTTAGGGAATTGTTAAGTCAAAACGAGTATGTATTATAACACATATTCTTCTAACCTAGCATTTTACATTAAATGG

>C15

CGTTTAGGTGGGAGCCATTAAGAACTTCGATTTGGAGCGCATGATGGGCTGTTGGCACGTGGTTCAGTATTACGCATCCACAGAGGAACTTCCGGAGTACGCGTGCATGCGCAGTCACTTTTCCTTTTCCAAAGAGGATCAGCATGTGAGTGGCAGCAAGGACCTTTATCCCTATTACAAATTATACCTCAATACCTCAATTTAAATTAGATAACGATGAACTTTAGCTACATATTTGCGGAGGATCCGCTGCGCGAGAAGCTCGTGGGCAACATCACCTGGATGATACCCAAATTCCAAGAGCCAGGACATTGGCAGCACACGGAGGACATATGTGAGTGATGGAAATGTTTCTACAATCAAATTCAAAACGAAAATCTATTCAAAATGTAGTTGTGATACGTTTGAAGTAGTTTGGTAATTACTATGCCAAAGAGTAATTCGATCTGCCC

>C16

TCTTGAGAAGATAAGGATAGAAACCAATAACACATAATAAAATATACTTTTACAGCCACATAGCTACATAAGATACAAGTTAGTGAATATATTATAAAATTGTCTAGACTACAGTATTTAGATTATCTCATGATATACCCGTAAGACTATCGTACACTTCCGGATCCATAGCGGATTTCCCTTTCGAACTGCTCCGCCTTCAAGGTGCCGTCTATACTCTCGCAATTCTTGTTGAAAACGCTGTAGGCACTCATCTCGTTCCGTTTCTTTGGCTCCGTTCGGGTGTTGAAGTAGATGCCAAAGAGGGCGGAAAACATGAGGAAAACCAATCCAAAGCTTAGCTTTATGGCAATAACGTAGAGAGTTATCCAGAACAGCAGATAGACAGTCCAGAGGGTGTACTTTAGGCAGTTGTTTTCCTTGTGGTGATGATCTGTGGATCTG

>C17

AAGACAAGTTTAAACAGCCTACGATTATGTTAACCATTCTAAGAAGATTCGCTAAGAAGTAGTGCGATTTATATGACCAACTGCTATCAAAATTTAGATGATTTTTTTAGTGTTGCAGTGAAATATTATACGACTTTGCTTTGCTTTTTCGGTGGAATAAATAGCCGGCCGGCTTGGCGGAGTTGGATGCCCACGCCCAAGCGGCATGTCCCATTCGGATACCCCATCCTGGAGTAGGCACCCGAAAATTGTACCGCTCTTGCCACATTTGGAGAGACGATCACGCTGCTGATATCATATTTTAGAGAGCATCATGCCCATTTTGGCAATGGCAAAACAGGCCGCTACACTACGGTCGGGCTCCCATAAGTAGAACGTCTTAAAGGTGCTCAATCTGCCATCAGATACATTGTTGCGCACAATGTACGCTCGATAGTCGAAGTTCTGGCTCCCCTTTCT

>C18

AACGCCGACTTGCTTAAAAAAACCTTTTCTATTAAATAGGAAACCAAGCTGGCAATGATCCTAAGCTTAAATAACCTTTTTTTTAGAGAGATCAAGAGACGCAAGAGTATAGATTTATAAGAATTAAGATGCATTGGAATACTATATGTGAATAATGGGAACTTGATGGATGGTTAGCTACCTTGAAGACCTCCTGGAGGGAGTAGACCAGCTCGGGACCCACACCGTCGCCGGGAATCAGTGTGCAGGTGGTTCTGTTGGCGCCGTAGTTATCCTGCAAGACCAATTAGTTTCCGGTTACAACTGGTGCCACTTAATAAGCGCATTTAACACTCACCGTAGCCCGTAGCGTCGA

>Positive Control

GAGCCCGGCTCTATTCACACTTTCTTTCGAACCGAGTCGGCCAGACTAGACCGTCGTATTATTGTGATTTACTTGAGCCGCTTTATCGCCGTAATCACAACAAAGTTGTGCGACAAATACAGATATGGGGACAAACAATAAGAGCGAGAGTTGCGGATGGCAGGCAGCTGCTTGGCTCCCAACCAACTAGAAAGTGACTAATCGGAGCTTGATGTCATTCCGGGACTCTGACCGGAAGACGACGGTACGCCGGGGCATTCGTTTAGGTCAACTAATTGCGACGTGTTTACACACTATCACAGTGCCAGGGTCTAAATCTGCGATGCAAATGGGTTCCCTTGCCAATGGCACGCTCGCTCATTGAGACATCAAATACAAGTTGACGAAAAGCGAGTATGACGTTTATTTAATTCGGAGTAATGATTTCATCGCTCACCGTGTAATCGATAAGCATCTACAGCAGGGTGCACGGAGGCAAACATAGTCCTGGAAGGGCGA

>Negative Control

TGAAAGTCGAGGAATTCAACCATAGTGTTCTCTCTTGCTCTAAAAATGTTAATTATGACATAGTCTAAAGCCTAGCTAGTTGGTAAATAACTTGCTGCTTTAAGCACTCAATATTTCGCTTACCATTTCGCTTGAGTCAACTGAAGTTCTTTGGCTAA

TTGCCGACAGAGTTCATTACCAATGCCCTGCAACTTTCTTAAGTGCTACATATGTTTGTGCATTTATGCCAGTCTCATTAGCATAGTGATATTGATTTTCTGGTTAACCATCAATCAACAAAACGTTAGACACACAGGCCCCAGATTGCTATGCAATTGAGGCTCCAAACCGCAGCACGCCATGCTCGAATGCCTTAAAGCGCCTGTTGGCATGCAGCAATTTATGTGCACATATCTGTGAGTGTGTGTGTGTGTGTGCAGAAGCTCCAAGGATGCGTGGCTGTGGATCCTTCGCCCAGAAGGGAGCAGCGCTGGAGGAAAAGCCAAATTCGTAGCTCGAT
